# Strategies to Broaden the Applications of Olive Biophenols Oleuropein and Hydroxytyrosol in Food Products

**DOI:** 10.3390/antiox10030444

**Published:** 2021-03-13

**Authors:** Mariana Monteiro, Andreia F. R. Silva, Daniela Resende, Susana S. Braga, Manuel A. Coimbra, Artur M. S. Silva, Susana M. Cardoso

**Affiliations:** LAQV-REQUIMTE, Department of Chemistry, University of Aveiro, 3810-193 Aveiro, Portugal; marianaicnamonteiro@gmail.com (M.M.); afrs@ua.pt (A.F.R.S.); danielaresende@outlook.pt (D.R.); sbraga@ua.pt (S.S.B.); mac@ua.pt (M.A.C.); artur.silva@ua.pt (A.M.S.S.)

**Keywords:** olive phenols, delivery, encapsulation, complexation, emulsions, chemical functionalisation, food application

## Abstract

Oleuropein (OLE) and hydroxytyrosol (HT) are olive-derived phenols recognised as health-promoting agents with antioxidant, anti-inflammatory, cardioprotective, antifungal, antimicrobial, and antitumor activities, providing a wide range of applications as functional food ingredients. HT is Generally Recognised as Safe (GRAS) by the European Food Safety Authority (EFSA) and the Food and Drug Administration (FDA), whereas OLE is included in EFSA daily consumptions recommendations, albeit there is no official GRAS status for its pure form. Their application in food, however, may be hindered by challenges such as degradation caused by processing conditions and undesired sensorial properties (e.g., the astringency of OLE). Among the strategies to overcome such setbacks, the encapsulation in delivery systems and the covalent and non-covalent complexation are highlighted in this review. Additionally, the synthesis of OLE and HT derivatives are studied to improve their applicability. All in all, more research needs however to be carried out to investigate the impact of these approaches on the sensory properties of the final food product and its percussions at the gastrointestinal level, as well as on bioactivity. At last limitations of these approaches at a scale of the food industry must also be considered.

## 1. Introduction

**Figure 1:**
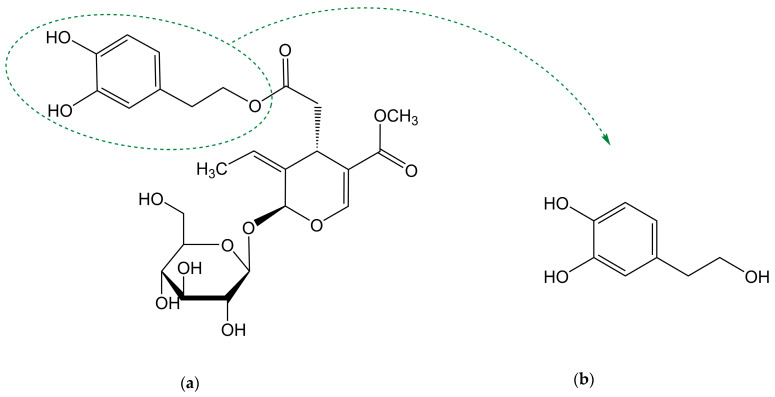
Chemical structure of (**a**) oleuropein and (**b**) hydroxytyrosol.

Biophenols derived from olives are used as traditional remedies for a variety of conditions, including inflammatory states and cardiovascular diseases. Oleuropein (OLE) and hydroxytyrosol (HT) are the most well-known compounds of this family. OLE is the main phenol in olive-derived olive products, being composed of an elenolic acid linked to an *o*-diphenol, hydroxytyrosol, and a glucose residue (Figure 1a). It is present in olive tree leaves and drupes. Its aglycone form is also found in olive oil (oleuropein is not soluble in oil due to the superior polarity) [1]. HT (Figure 1b), also known as 3,4-dihydroxyphenylethanol (DOPET) or 3,4-dihydroxyphenolethanol (3,4-DHPEA) or 4-(2-hydroxyethyl)-1,2-benzenediol, results from OLE degradation. It is found mainly in olive oil and cured olives and, in lower amounts, in olive leaves and other products such as grapes, wine and olive by-products [2,3,4]. Its occurrence in wine has been proposed to result from alcoholic fermentation or from the enzymatic oxidation of tyrosol (TYR) [2,5]. OLE is responsible for the bitter taste of fresh olives. To become acceptable by consumers, fresh olives are submitted to a de-bittering process in which OLE is hydrolysed to HT and elenolic acid, two non-bitter compounds [6]. Degradation of OLE occurs both during the maturation of the olives and oil storage [7].

The amount of these compounds in their natural sources is variable. A database on polyphenol content in foods reports that OLE average content in olive oils varies from 0.17 mg/100 g for extra virgin oil to less than 1 µg/100 g for virgin ones, while in black and green olives its mean content is 72 and 56 mg/100 g [8]. On the other hand, the average content of HT in olives and derivatives varies, in mg/kg of product, from 3.5 in virgin olive oils, to 7.7 in extra-virgin olive oils, 659 in black olives and 556 in green olives [9].

The antioxidant activity of OLE and HT is, as observed for other phenolic compounds, dictated by the presence of an *o*-diphenolic group in their structures. Such fact was corroborated by many authors through in vitro and in vivo studies [10,11]. These compounds are also claimed to exert anti-inflammatory, cardioprotective, antifungal, antimicrobial and antitumor activities, and to have potential physiological benefits on plasma lipoproteins, platelets and cellular function [12]. Moreover, HT is also mentioned to contribute to bone health [7]. 

The claimed health-promoting properties of OLE and HT, allied to their easy and affordable recovery from olive by-products, make them excellent ingredients for application in functional foods [13]. HT is Generally Recognised as Safe (GRAS) by the European Food Safety Authority (EFSA) and the Food and Drug Administration (FDA) and claimed to benefit human health when ingested at a daily dose of at least 5 mg (this includes related compounds such as OLE) (EC No. 432/2012) [14,15]. Moreover, HT is authorised as a novel food ingredient by the European Commission (EC) under the regulation EC No 258/97 of 2017 [16]. OLE is included in EFSA’s daily consumption recommendations, albeit there is no official GRAS status for its pure form. Notably, an olive leaf extract Bonolive^®^, containing 80% OLE, was approved for use as an ingredient in a range of food categories, including yoghurt, confectionery, fine bakery wares and beverages (EC No 258/97), and received Self-affirmed GRAS status in the United States in 2016 [17]. Note that Self affirmed GRAS status refers to an independent GRAS determination not involving the FDA. In this status, the stakeholder is not legally obligated to notify the FDA of its determination before adding the substance to food, but may voluntarily submit their independent GRAS determination to the FDA for review and response [18]. These facts support the use of OLE as a food ingredient.

Since the amount of OLE and HT obtained through their natural sources’ consumption is far below the recommended daily intake, it is important to promote their intake by incorporating them in other types of foods. Examples include beverages, fish-based products and vegetable oils, as already reported for OLE [19] and for HT [20,21].

The use of OLE and HT as a food ingredient at the industrial scale faces various challenges. OLE is unstable in water or ethanol if under UV light, decomposing through hydrolysis or transesterification [22]. Moreover, its application often causes sensorial constraints due to its bitter taste, requiring the use of taste-masking compounds such as sodium cyclamate and sucrose [23]. HT is highly sensitive to air and light due to its hydroxy groups and its amphiphilic character brings difficulties in mixing it into foods. Additionally, HT may interact with proteins and other food nutrients, which may affect the extension of the metabolisation and, therefore, its bioavailability in the body [24]. Thus, the food matrix must also be considered when aiming to use OLE and HT as food ingredients. The following sections highlight relevant strategies that have been tried by distinct authors to overcome such challenges, particularly the encapsulation in delivery systems and the synthesis of OLE and HT derivatives.

## 2. Encapsulation of OLE and HT

Encapsulation is a technique in which one or more ingredients (active material or core) are trapped within some form of a matrix (wall, encapsulating agent, shell or carrier material) that can vary in form and size, including solid or liquid, homogeneous or heterogeneous and microscopic or macroscopic [25]. Nanoencapsulation often refers to capsules with dimensions on the scale of 1–1000 nm, although some authors usually consider those with dimensions above 100 nm as microcapsules [26]. The maximum size limit for microcapsules is more consensual, being generally considered at 800 µm [27]. The wall material of these capsules may include lipids, proteins, hydrocarbons, or other polymers [28].

Olive-derived biophenols have been encapsulated in different types of delivery systems with a variety of purposes: protecting them from degradation, increasing solubility in particular media, allowing their controlled delivery, and enhancing their bioavailability. It is unquestionable that of the two olive biophenols, HT is by far the most investigated, possibly not only due to its antioxidant power and tasteless character, but also as a product of OLE degradation (a favorable aspect to obtain this compound in considerable amounts).

### 2.1. Lipids

The main advantage of using lipids as components of nano and microcapsules is that they are biocompatible and biodegradable [29]. Liposomes are among the first lipid nanoparticles ever developed and they result from the self-assembly of the amphiphilic phospholipids in an aqueous medium into one or several concentric closed bilayers. Relevant studies focusing on the application of these structures to olive biophenols are summarised in Table 1.

OLE was shown to have superior encapsulation efficiency (30%) in liposomes than HT and tyrosol (TYR) (12% and 4% respectively), with improved physical stability and less tendency to aggregate [30]. In addition, no cytotoxic effects were noticed for the encapsulated OLE and HT on human chondrocyte cells cells at concentrations tested: max 1.4 × 10^−1^ and 6.0 × 10^−2^ mM, respectively [30]. Still, Yuan and coworkers [31] were able to encapsulate HT in nanoliposomes (average size of 200 nm) with a 45% efficiency. The authors highlighted the enhanced stability of encapsulated HT in aqueous media, which increased from 7 to 30 days, at 25 °C, allowing its prolonged release without any loss of α-diphenyl-β-picrylhydrazyl (DPPH) radical scavenging activity. In another study, Evans and Compton [32] produced liposomes enclosing an HT derivative, namely phosphatidyl-HT, at a subnanomolar range of concentrations. The liposomes had a size of approximately 85 nm, a surface charge of −25 mV, and they were suggested to afford formulations with good stability. Moreover, OLE was encapsulated in 1,2-dipalmitoyl-*sn*-glycero-3-phosphocholine (DPPC) liposomes and interaction studies showed that OLE can strongly interact with phospholipid headgroups. Additionally, in the same study, olive leaf extracts rich with OLE were encapsulated in DPPC liposomes with a mean encapsulation efficiency of 34% and a mean particle size of 405 nm, which show good stability in commercial lemonade drink over long periods (47 days) at refrigeration temperatures (5 °C) [33]. Another study used ufasomes (unsaturated fatty acid liposomes made up of oleic and linoleic acids) to encapsulate OLE. The in vitro studies on CaCo-2 cells demonstrated the great ability of ufasomes to interact and to be internalised into cell model and the improvement of natural antioxidant activity of OLE against oxidative stress induced by H_2_O_2_ on cell model [34].

**Table 1:** Reports of lipid-based encapsulation systems for Oleuropein (OLE) and hydroxytyrosol (HT).

Formulation	Application	Main Findings	Ref.
Liposomes			
Liposomes with OLE, HT and TYR	Drug-delivery system	↑ EE% for OLE No cytotoxic effects on human chondrocyte cells	[30]
DPPC liposomes with OLE	Beverages	EE: 34% Particle size: 405 nm Stable in commercial lemonade drink over 47 days at 5 °C	[33]
Ufasomes with OLE	Claim for food application	↑ antioxidant activity of encapsulated OLE against oxidative stress induced by H_2_O_2_ on CaCo-2 cells	[34]
Liposomes with phosphatidyl-HT	Claim for food application	Particle size 85 nm; Surface charge: <−25 mV (stable liposomes)	[32]
Nanostructured lipid carriers			
OLE-loaded NLC	Claim for food application	OLE leakage was not observed in the nanocarriers within the 3 months of storage Good stability of OLE-loaded NLC	[35]
Emulsions			
Lipid emulsions and microemulsions	Claim for food application	Digestibility assay: ↓ Gastric lipolysis of microemulsion compared to emulsions. ↓ Effect of duodenal lipolysis by the dispersion type.	[36,37]
OLE-loaded W/O/W	Claim for food application	Emulsions were stabilised for + than 40 days of storage with ↑ hydrophobic emulsifier concentration and ↓ OLE concentration	[38]
OLE-loaded O/W	Claim for food application	Stable monodisperse oil-in-water O/W was produced when higher hydrophobic triglyceride oils are used	[39]
OLE-loaded O/W	Claim for food application	↑ stability due to the surface activity of OLE	[40]
Nano OLE-loaded W/O/W	Claim for food application	Optimum conditions for formulation: 8% WPC, 1.97% pectin and 8.74% Span 80 EE: 91%; Droplet size: 191 nm; Surface charge: −26.8 mV	[41]
O/W, W/O/W and GDE with HT and perilla oil	Claim for food applications	Emulsions structurally stable at 4 °C up to 22 days. HT losses up to 24% throughout the storage of GDE → ↓ antioxidant activity of the emulsion. No lipid oxidation during storage.	[42]
GDE with HT	Animal fat replacing	Physical properties: ↑ formation of weaker gels; no significant loss levels until 30 days; minimal changes in colour and pH of W/O/W during storage. Oxidation: systems little prone to oxidation even at 30 days. Biological activity: ↑ antioxidant and ↑ antimicrobial activity	[43]
HT in W/O/W enriched in chia oil	Meat supplementation	Presence of HT: ↑ oxidative stability: ↑ DPPH free radicals scavenging; ↑ FRAP; ↓ TBARS	[44]

EE—Encapsulated efficiency; DPPH—α-diphenyl-β-picrylhydrazyl; GDE—Gelled double emulsion; FRAP—Ferric reducing antioxidant power; HT—Hydroxytyrosol; NLC—Nanostructure lipid carriers; OLE—Oleuropein; O/W—Simple emulsion; and TBARS—Thiobarbituric acid reactive substances; TYR—Tyrosol; W/O/W—Double emulsion.

Solid lipid nanoparticles, which contain lipid in the solid stage at room and body temperature, have some limitations such as leakage of encapsulated compounds. This lead to the development of nanostructured lipid carriers (NLC), in which the lipid phase contains both solid (fat) and liquid (oil) lipids at room temperature [45]. Soleimanifard et al. [35] demonstrated that OLE-loaded NLC was stable for a period of 3 months, without leakage from the nanocarriers when kept at −18 °C and room temperature.

**Figure 2:**
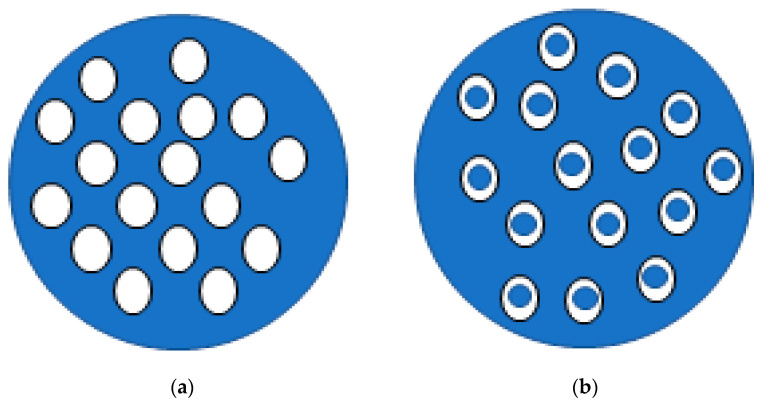
Schematic representation of simple (**a**) and double (**b**) emulsions: white fill correspond to the oil phase and blue fill represents water phase.

Lipids can also be used in the formulation of microemulsions. Conventional emulsions (often having micro and macrosized droplets) are dispersions of one liquid phase into another liquid phase (immiscible), forming droplets with high interfacial tension that are, therefore, thermodynamically unstable. In turn, lipid microemulsions (emulsions with microscopic droplets) are known to be thermodynamically stable mixtures formed by an aqueous phase, an oil phase and a surfactant [46]. They differ from other lipid particles due to the absence of a solid lipid in the oil phase [47,48], and can be organised as simple or double emulsions (W/O/W) (Figure 2). Double emulsions, in which the oil droplets are dispersed in the aqueous phase, also contain smaller water droplets in their interior [49]. In general, these are more suitable for the encapsulation of compounds with high hydrophilicity, avoiding the loss of compound to the continuous phase [50,51]. Some studies are listed in Table 1.

Chatzidaki et al. [36,37] developed a microemulsion (droplet size of 20 nm) and an emulsion (droplet size of 354 nm) for HT encapsulation, having found that both systems were able to keep the high antioxidant activity of this compound. The authors also noted that the microemulsion was more resistant to gastric digestion, probably due to lower gastric lipolysis.

W/O/W emulsions have also been used to encapsulate OLE and HT. Souilem et al. [38] produced food-grade W/O/W loaded with OLE, noticing that it only remained stable (for 40 days) for low loading ratios of OLE (0.1–0.3 wt.%). Later, the same authors demonstrated that due to OLE’s interfacial activity, it was possible to produce stable oil-in-water (O/W) emulsions in high hydrophobic triglyceride oils, such as refined soybean oil, extra virgin olive oil and refined olive oil [39]. Further, through molecular dynamics studies, these authors also proved the influence of the surface activity of OLE in the preparation, stability and delivery of the food emulsions. The amphipathic character of OLE decreased the interfacial activity between oil and water phases, showing an emulsifying ability that stabilizes O/W droplets [40]. On the other hand, Gharehbeglou et al. [41] produced double nano-emulsions with OLE stabilised by pectin–whey protein concentrate (WPC) complexes, having set the optimum conditions for this formulation as 8% WPC, 1.97% pectin, 8.74% Span 80 (sorbitan mono-oleate, a non-ionic surfactant), 1:4 ratio of inner-to-outer phase and pH 6.1. The obtained nano W/O/W was characterised by a droplet size of 191 nm, zeta potential of −26.8 mV, and encapsulation efficiency of 91%. 

HT was also previously shown to be loaded in W/O/W, albeit less retained than in simple systems, according to Flaiz et al. [42]. In addition, gelled W/O/W, i.e., W/O/W gellified at 4 °C for 24 h, offers an interesting possibility for the food industry to encapsulate bioactive compounds and provide certain plastic properties. Although the formation of gelled W/O/W (in this case, gelled by 4% gelatin and 2% transglutaminase) is advantageous in decreasing phase separation, the HT losses were still above those of simple emulsions during storage [42]. Nevertheless, the same research group showed that gelled W/O/W with HT were stable for up to 30 days and could be used as a healthier replacement for meat fat [43], allowing to retard microbial growth and oxidation. In addition, Cofrades et al. [44] reported that W/O/W of chia seed oil and perilla oil with HT were highly stable. When used to supplement meat at 100 mg/kg meat, this microemulsion system improved the antioxidant properties of the supplemented food products, and simultaneously reduced their oxidation levels. This tendency was even more evident when a simple HT emulsion was used [44].

### 2.2. Biopolymer-Based Systems

The production of colloidal delivery systems with food-grade biopolymers such as polysaccharides and proteins is an affordable and practical strategy for encapsulation in food applications. The encapsulant material may vary in composition (protein and/or polysaccharide type), structure (homogenous, core–shell and dispersion) and dimensions, thus achieving distinct systems categories, including biopolymer nanoparticles, hydrogel particles and filled hydrogel particles [52]. As with other systems, most authors focused on HT instead of OLE (Table 2).

**Table 2:** Reports of biopolymer-bases systems on the delivery of OLE and HT: cellulose, starch, pectin and biocomposites.

**Formulation**	**Application**	**Main Findings**	**Ref.**
Cellulose microcapsules with HT	Claim for food application	EE: 82.4–88.1% Particle size: 156.6–304.0 µm Microcapsules with HT are gastro-resistant and retain > 50% of their antioxidant capacity in simulated GI fluids.	[53]
Starch granules with HT and probiotics	Nutraceuticals	Resistant against GI tract conditions and stable up to 6 months of storage under refrigeration. ↓ HT bioavailability by the administration of live *L. plantarum* bacteria with the olive phenol-containing extract, compared to the extract alone.	[54]
Starch nanocrystals or nanoparticles in a PVA film with HT	Active packaging	HT migrated values for all formulations ≤ migration limits for food contact materials. Gradual release of HT during 21 days. Highest gradual release for films with starch nanoparticles. ↑ antioxidant activity for all ternary formulations over time.	[55]
Poly(ε-caprolactone)-based NC and montmorillonite, Cloisite30B films with HT	Active packaging	HT ↑ poly(ε-caprolactone) crystallinity, ↓ thermal stability and plasticizing effect. Interaction of HT-Cloisite30B led to a prolonged release of the HT.	[56]
Pectin plus fish gelatin composite films with HT and DHPG	Strawberry preservation	↑ stretching capacity and resistance to breakage. The edible film preserved strawberries with a significant delay in visible decay.	[57]
	Meat preservation	↓ lipid oxidation in raw beef meat during refrigerated storage. Film with adequate mechanical and oxygen barrier properties. Film with beeswax ↓ lipid oxidation and ↓ the oxygen barrier capacity.	[58]
MD-OLE and IN-OLE	Claim for food application	Protection of OLE from GI conditions.	[59]
Eudraguard^®^ protect with HT	Claim for food application	Spherical non-aggregate particle (particle size: 230 nm) Loading capacity of HT: 38%	[60]

DHPG—3,4-dihydroxyphenylglycol; EE—Encapsulated efficiency; GI—Gastrointestinal; HT—Hydroxytyrosol; IN—Inulin; MD—Maltodextrin; MF—multifunctional; NC—Nanocomposite; OLE—Oleuropein; PVA—poly(vinyl alcohol).

Owing to their neglectable toxicity and high biodegradability, cellulose and its derivatives have been extensively used for compound delivery in several fields [61,62,63,64]. Paulo and Santos [53] tested the use of ethyl cellulose to provide HT in polymeric microcapsules (mean particle size 156.6–304.0 µm), concluding that the capsules remained stable over a wide temperature range and had good encapsulation efficiency (≈88%, regarding the proportion of HT encapsulated *versus* the amount added) despite a low loading of 5% (ratio between the amount of encapsulated compound and amount of vehicle). In addition, the authors demonstrated that the formulated capsules were gastro-resistant and retained over 50% of the initial amount of HT’s antioxidant activity in simulated gastrointestinal fluid [53].

Starch granules and nanostructures were investigated as alternatives to cellulose for encapsulation of compounds in food applications. Aponte and colleagues [54] formulated starch granules for the combined delivery of HT and probiotics, highlighting their stability and gastro-resistance, as well as the ability to improve the amount of bioavailable HT. Moreover, HT was incorporated in starch nanostructures and applied in active packaging films of poly(vinyl alcohol) (PVA), allowing the gradual release of HT while preserving its antioxidant properties [55]. The same type of application was also achieved by Beltrán et al. [56], in poly(ε-caprolactone) and Cloisite 30B films containing 5–10 wt.% HT. The presence of Cloisite 30B decreased the release of HT, while the presence of HT improved crystallinity but decreased thermal stability, acting simultaneously as plasticizing [56].

A composite film made with pectin and gelatin with HT and 3,4-dihydroxyphenylglycol (DHPG) was applied for the preservation of strawberries [57] and raw meat [58]. Notably, these films had superior stretching capacity and resistance to breakage in comparison with films containing no olive-derived biophenols. Furthermore, the films effectively delayed both mould growth and lipid oxidation on the food products, for at least 7 days of storage. Maltodextrin (MD) and inulin (IN) are also used as delivery systems for bioactive compounds. MD, a saccharide that contains D-glucose units linked with α-(1→4) glycosidic bonds, has high solubility in water, low viscosity, and it forms colourless solutions. Moreover, it is a very digestible polymer from which bioactive compounds may be quickly released during digestion [65]. In turn, IN, a branched fructo-oligosaccharide composed of β-(2→1) glycosidic linked fructose units, can pass relatively intact through the gastric tract [66]. To our knowledge, the application of MD and IN to deliver olive-derived biophenols is yet rather rare to date. Even so, as pointed by González et al. [59], MD-OLE and IN-OLE encapsulations allow protecting OLE from gastrointestinal conditions and it significantly improves their bioaccessibility (15% and 12% for MD-OLE and IN-OLE, respectively, vs. 1.5% in non-encapsulated OLE). The food-approved-biopolymer Eudraguard^®^ protect (composed of methacrylic acid and methyl methacrylate monomer units) was used to encapsulate HT-rich olive oil by supercritical fluid extraction of emulsions, leading to the formation of spherical, non-aggregated particles of an average size of 230 nm and resulted in HT-oil loads of 38% [60].

### 2.3. Complexation Methods 

Molecular inclusion is a process in which a “host” molecule has a cavity into which a “guest” molecule can be accommodated to form an inclusion complex [25]. Cyclodextrins (CDs) are the most commonly used host molecules in foods due to their GRAS status by the FDA. A few studies report CDs as hosts for OLE or HT inclusion (Table 3) [67,68]. CDs, obtained from the enzymatic degradation of starch [67], are macrocyclic oligosaccharides having 6, 7, or 8 α-D-glucose units, being called, respectively, α-, β-, and γ-CD [69]. CDs have a hollow, truncated conical shape and are the encapsulating agents of choice to improve the stability, activity and solubility of various bioactive compounds [70]. 

**Table 3:** Reports regarding the molecular encapsulation of OLE and HT.

Formulation	Application	Results	Ref.
Oleuropein			
α-CD·OLE, β-CD·OLE and Ɣ-CD·OLE	Claim for food application	OLE form binary complexes (1:1) with the three types of CDs β-CD is the most effective for complexation.	[71]
β-LG·OLE	Claim for food application	↑ stability of formed complexes and validity of docking results for β-LG·OLE.	[72]
OLE·ALA	Claim for food application	OLE binds to ALA mainly via electrostatic, van der Waals and hydrogen bonds.	[73]
Hydroxytyrosol and Oleuropein			
β-CD·HT, β-CD·OLE and β-CD·TYR	Claim for food application	No OH group of HT and OLE is shielded in the β-CD cavity Antioxidant activity: β-CD·HT > β-CD·OLE > β-CD·TYR.	[74]
Hydroxytyrosol			
β-CD·olive biophenols	Claim for food application	↓ bitter taste and preserves them against chemical and physical decomposition reactions during storage.	[75]
β-CD·HT, HP-β-CD·HT	Claim for food application	Insertion of the HT through the narrower face of the CDs. ↑ antioxidant capacity and photoprotection of HT.	[76]
β-CD·HT	Food industry	↓ HT bioaccessibility (−20%) and absorption (−10%) in presence of foods (7 mg of HT in the meal). β-CD did not affect bioaccessibility and absorption.	[77]
β-CD·HT	Claim for food application	β-CD and drying processes do not affect the efficiency of HT to reduce the DPPH radical.	[78]
HT/DHPG-soluble and insoluble dietary fiber of apple cell wall	Dietary fiber	Non-covalent interaction between phenols and the apple cell wall fibers. Antioxidant activity of HT/DHPG was not altered after complexation with apple cell wall fibers and after a simulated gastrointestinal digestion.	[79]

ALA—α-lactalbumin; CDs—Cyclodextrins; DPPH—α-Diphenyl-β-picrylhydrazyl; DHPG—3,4-Dihydroxyphenylglycol; HP-β-CD—2-(Hydroxy)propyl-β-cyclodextrin; HT—Hydroxytyrosol; OH—Hydroxy group; OLE—Oleuropein; TYR—Tyrosol; α-CD—α-Cyclodextrin; β-CD—β-Cyclodextrin; β-LG—β-Lactoglobulin.

Structure analysis of complexes between OLE and CDs (OLE·CD) has been studied by Efmorfopoulou et al. [71], who proved that OLE forms binary systems (1:1) in neutral aqueous solutions with the three types of CDs and that β-CD was the most effective for complexation. Proteins such as β-lactoglobulin (β-LG) and α-lactalbumin (ALA) were also investigated regarding their interaction with OLE [72]; these proteins have the advantage of providing desirable textures in food. Vanaei and co-workers studied the formation of a complex between β-LG and OLE by molecular docking, having shown its high stability [72]. In the case of OLE·ALA complexes, Katouzian et al. [73] revealed that OLE binds to ALA mainly via electrostatic, van der Waals interactions and hydrogen bonds, as confirmed by Fourier-transform infrared spectroscopy (FTIR), fluorescent spectroscopy, dynamic light scattering, circular dichroism spectroscopy and molecular docking analysis.

**Figure 3:**
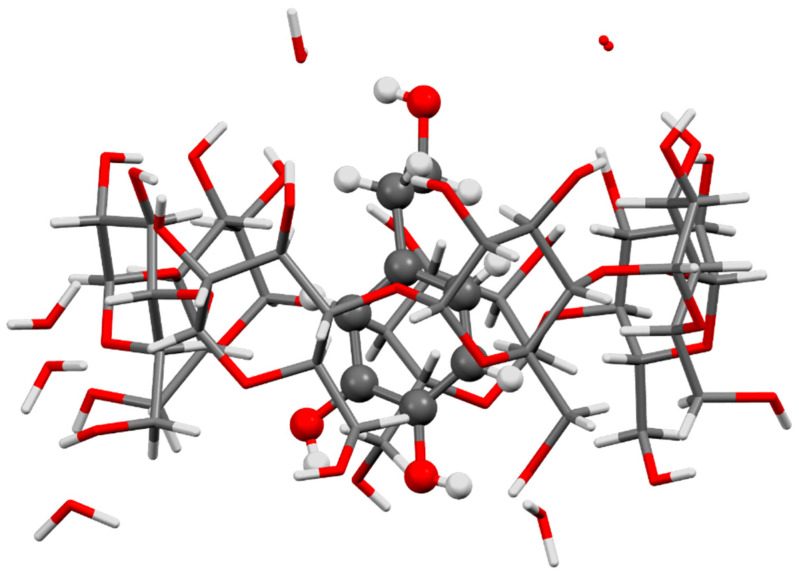
Structure of the inclusion complex β-CD·HT·6H_2_O, reported by Aree and Jongrungruangchok from single-crystal X-ray diffraction studies [74]. A single unit of the complex is represented, along with the six hydration waters one of them with disorder; the molecule of HT is represented with the ball-and-stick model for highlight, showing the catechol hydroxy groups facing downwards to the smaller rim of the host. Waters and the β-CD molecule are represented by sticks. Image was redrawn with the software Mercury 3.5.1 (Copyright CCDC 2001–2014) from the atomic coordinates of β-CD·HT·6H_2_O, available from the CCDC with the refcode CIQFOA.

Inclusion of HT for antioxidant and food applications was studied mainly with β-CD and, less frequently, with its (2-hydroxy)propylated derivative, HP-β-CD (Table 3). Solution studies conducted by Rescifina et al. focused on the thermodynamics and geometry of the interactions of β-CD with various phenolic components of olives, which included HT and other phenols (tyrosol, homovanillic acid, protocatechuic acid and 3,4-dihydroxyphenylacetic acid) [75] aiming at decreasing the bitter taste of these compounds and at increasing their stability to light, heat and oxidation. All the studied phenols showed inclusion stoichiometry of 1:1, that is, one molecule of β-CD was able to include one molecule of the phenol in its cavity; the host–guest affinity was determined by measuring the association constants (K_1:1_) at 20 °C, which ranged from 137 (for homovanilic acid) to 661 M^−1^ (for protocatechuic acid); for HT, the affinity value was in the middle of the range, at K_1:1_ = 335 M^−1^. The authors further postulated that the compounds with a strong host–guest affinity had a low bitterness degree, since they remain in the cavity and thus are less available for interaction with tastebuds. López-García et al. [76] further confirmed 1:1 stoichiometry for HT interaction with β-CD in aqueous solution, but obtained [also from nuclear magnetic resonance (NMR)] a lower estimation for K_1:1_, around 41 M^−1^. The authors also investigated HT inclusion into HP-β-CD and postulated, for the two cyclodextrins, an inclusion geometry with the catechol hydroxyl groups of HT would face the wider rim of the CDs, which would make them easily accessible and thus explain the observed 25% increase in DPPH scavenging activity. Excess of HP-β-CD and of β-CD (4:1) was further shown to protect HT from degradation by UV light [76]. Notably, the crystal structure of β-CD·HT·6H_2_O, reported by Aree and Jongrungruangchok [74], established the definitive geometry as being rather the inverse, with the catechol group of HT facing the narrow rim of the host (see Figure 3). The narrow rim is the one with primary hydroxy groups and thus a much more flexible aperture to the cyclodextrin’s cavity (compared to the wide rim), allowing included HT molecules to react and perform their radical scavenging activity. These authors also observed an increase in the DPPH scavenging activity of β-CD·HT in comparison with pure HT, which was of *ca* 11% at the tested conditions. 

Malapert et al. [78] evaluated the influence of the preparation method on the properties of the inclusion complexes of β-CD·HT (1:1 proportion). Two methods suitable for large-scale industrial production were chosen, freeze-drying and spray-drying (with a spray head temperature of 85 °C). The presence of inclusion complexes in the two solid products was confirmed by solid-state NMR and, most importantly, the DPPH radical scavenging ability of HT was preserved in the solid products (even through the heat of the spray-drying process) [78]. The freeze-dried β-CD·HT complex was also shown to have similar in vitro bioaccessibility and bioavailability to pure HT in an aqueous matrix or as an ingredient in foods (represented by a test meal containing pureed potatoes, minced beef and refined olive oil) [77].

Complexation methods also resort to non-cavitand molecules, such as pectin and other fibers. In this case, complexation occurs through non-covalent interactions and it differs from inclusion because there is not a defined cavity in the host molecule. Soluble and insoluble dietary fibers of the apple cell wall were used as hosts for complexation with HT, DHPG and a mixture of both. Results showed that HT/DHPG partially maintained their antioxidant activity after complexation with the fibers and after being submitted to partial fiber digestion in simulated gastrointestinal fluids [79].

### 2.4. Microorganisms

Microorganisms can also be used as carriers of bioactive compounds. This is a novel topic of investigation and the few studies reported so far investigated the capacity of *Saccharomyces cerevisiae* to adsorb phenolic compounds and protect them from digestion conditions [80,81]. Among them, Jilani et al. [82] adsorbed olive phenolics from leaf infusions into *Saccharomyces cerevisiae* and showed that the infusions did gain protection from degradation during gastrointestinal digestion when they were adsorbed in these microorganisms. Notably, the authors reported that the adsorption of these infusions increased the bioaccessibility, namely of OLE and HT, as well as antioxidant capacity, when compared with non-adsorbed infusions [82].

## 3. Chemical Modifications of OLE and HT

The preparation of carefully designed derivatives of OLE and HT can help curb their chemical stability issues and increase biological activity by altering the pharmacokinetic profile (i.e., the processes of absorption, distribution, metabolism and excretion) [83,84,85]. Two different approaches were used in the preparation of these derivates: chemical and enzymatic synthesis. Although chemical synthesis has been reported with satisfactory yields, enzymatic synthesis is more interesting for the food industry, since it affords natural products, avoiding the use of toxic reagents and enabling an easy purification of the product [86]. OLE can be converted to HT by acid and enzymatic hydrolysis [87] or even modified through acetylation and triazole derivation to improve its bioavailability. In addition, several works focused on the synthesis of HT derivatives, including esters, ethers, phospholipids, isochromans, polyacrylates, glycosides, selenium and sulphur-bearing analogues.

### 3.1. Modifications of OLE

**Figure 4:**
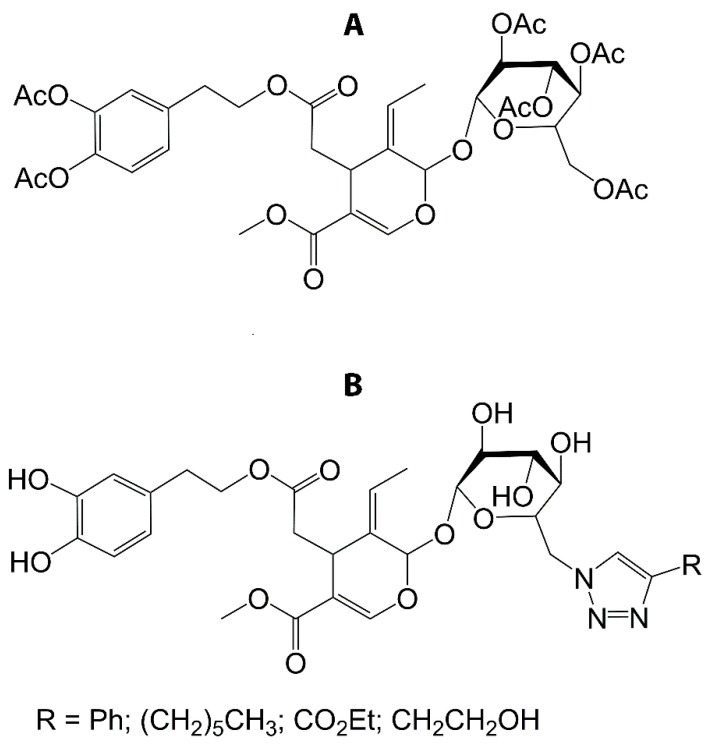
Structural representation of the derivatives of OLE resulting from acetylation (**A**) and tosylation (**B**).

Bonacci and coworkers [88] synthesised an acetylated derivative of OLE (OLE-Ac) with the purpose of improving OLE bioavailability from food, making it suitable for application in fatty foods. The authors demonstrated that OLE-Ac (Figure 4A), top) was stable for 24 weeks and its usage at 1:1 ratio (*w*/*v*) in olive oil showed no negative effects on the stability or sensory characteristics [88]. The presence of acetyl groups increases the lipophilic character of the molecule, thus allowing for increased permeability of the cell membrane [89]. Once inside the cells, deacetylation of hydroxytyrosol acetate occurs [90] and the antioxidant capacity of HT is regenerated. This may also explain the higher capacity of OLE-Ac than free OLE in breast cancer cell lines, MCF-7 and T-47D [91]. In another instance, Jerbi et al. [92] presented an efficient strategy of selective modification of the primary alcohol position of OLE that afforded triazole derivatives of OLE (Figure 4B) in good yields (≥82%).

### 3.2. Modifications of HT

**Figure 5:**
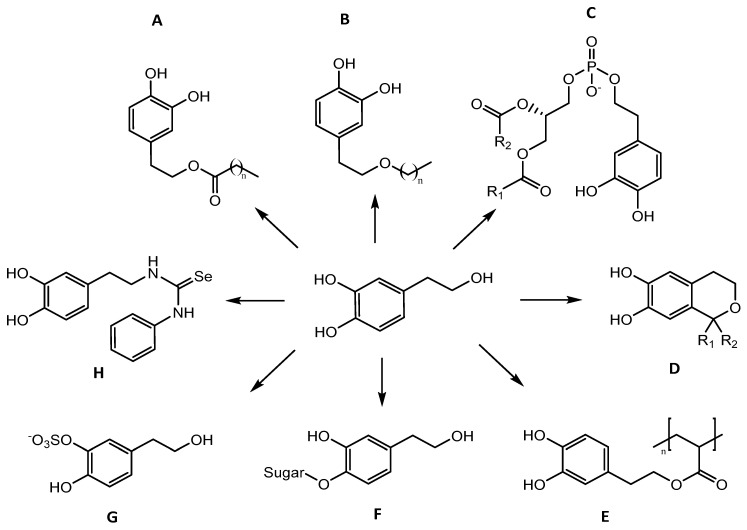
Structural representation of derivatives of HT: (**A**). HT esters; (**B**). HT ethers; (**C**). Phospholipids; (**D**). Isochromans (with R_1_ = H, R_2_ = 3,4-substituted phenyl or R_1_ = R_2_ = CH_3_); (**E**). Polyacrylates; (**F)**. Glycosides and (**G**). Sulphur and (**H**). Selenium derivatives.

Among the mentioned strategies to improve HT bioavailability and/or bioactivity, the synthesis of HT esters by reaction of its primary alcohol group with a fatty acid of variable length was the most explored (Table 4, Figure 5A). In general, these modifications improve the lipophilic character of HT without impacting the catechol moiety (*o*-dihydroxyphenyl group), thus preserving its antioxidant capacity, although this depends on several factors like the length of the alkyl chain [86,93].

**Table 4:** Reports regarding the derivation of HT claimed for food application: esters and polyacrylates.

Application	Results	Ref.
Virgin olive oil	↑ oxidation preventive action with HT.	[94]
Claim for Food application	The protective effects against DNA damage of HT esters were inversely proportional to their chain length.	[86]
Food application	Antioxidant capacity of HT esters > TYR esters. ↓capacity in lipophilic food matrices.	[93]
Functional foods	HT octanoate (C8) is the most effective to inhibit the oxidation in fish O/W.	[95]
Claim for food application	↓ antioxidant activity of HT esters with alkyl chain length around C8–C11 in different matrices	[96]
Claim for application	↓ Antioxidant function of HT esters with chain lengths > C10, measured through ABTS (in ethanol medium) and DCF (on cultured muscle cells).	[97]
Claim for food application	HT esters were produced by enzymatic transesterification with cuphea oil. HT esters antioxidant activity ≈ HT decanoate (C10).	[98]
Therapeutic strategy	Antioxidant capacity of HT laurate (C12) > HT against H_2_O_2_ induced apoptosis in U937cells and C2C12 murine myoblasts.	[99]
Claim for food application	Antioxidant activity of all the HT, with exception of HT stearate (C18), >HT C10 esters in human erythrocytes. C12 were optimum in scavenging free radicals.	[100]
Claim for food application	HT esters were able to scavenge DPPH radical, render inhibitory effects on cupric ion-induced LDL oxidation and show protective effects against hydroxy radical- and peroxy radical-induced DNA scission.	[101]
Claim for food packaging application)	PHTA (up to 50 mg/mL) fully scavenged DPPH free radicals. No cytotoxic activities from polyacrylates films in RAT1 normal fibroblast cells were observed for concentrations of 0.25–1 mg/mL of polyacrylate film	[102]

ABTS—2,2′-Azino-bis(3-ethylbenzothiazoline-6-sulfonic acid); DCF—Dichlorodihydrofluorescein fluorometric assay; DPPH—α-Diphenyl-β-picrylhydrazyl; DNA—Deoxyribonucleic acid; HT—Hydroxytyrosol; H_2_O_2—_Hydrogen peroxide; LDL—Low Density Lipoprotein; O/W—Oil-in-water emulsion; PHTA—Poly(hydroxytyrosyl)acrylate; TYR—Tyrosol.

#### 3.2.1. Esters

The amelioration of antioxidant abilities of HT by the formation of esters with fatty acyl chains varying from two (C2) to eighteen carbons (C18) (Table 4, Figure 5A) was reported by Trujillo et al. [94]. The authors demonstrated that, when incorporated in lipid matrix from virgin olive oil, HT esters had a more potent oxidation preventive action than pure HT and even than the commercial antioxidants butylated hydroxytoluene (BHT) and α-tocopherol. The same effect was demonstrated on protein oxidation, which was measured using a brain homogenate ex vivo model. Grasso et al. [86] further demonstrated that C2 to C18 esters of HT effectively protect DNA from H_2_O_2_-induced oxidative damage in samples of primary whole-blood cells, in a potency that was inversely proportional to their chain length. Moreover, in agreement with the “polar paradox” (a theory which indicates that hydrophilic compounds are more efficient in bulk oil than lipophilic antioxidants) [103], HT esters showed less activity in lipophilic food matrices than HT, as measured by a rancimat test [93].

Yet, Medina et al. [95] reported that among C2 to C18 HT esters, the C8 derivative was the most effective in preventing oxidation in fish O/W, which refutes the “polar paradox” theory. These conclusions were also in line with the study of Lucas et al. [96] on the surface-active properties of HT esters, which demonstrated that the antioxidant activity of HT esters increased when optimum hydrophilic–lipophilic balance range (around C8–C11) was attained. This is also consistent with the work of Tofani et al. [97] that revealed decreased antioxidant activity for HT esters with chain lengths greater than C10 in cultured muscle cells. Notably, Laszlo et al. [98] suggested that natural oils such as Cuphea oil could serve as a source of capric acid to produce C10 HT esters with high antioxidant potential, similar to those produced with synthetic fatty acids. 

Close tendencies on the above-described antioxidant potential of HT esters were reported by other authors using distinct experimental models. Burattini et al. [99] compared the previously selected three different classes of HT esters, namely with short (C2–C4), medium (C10–C12) and long (C16–C18) acyl side chains and showed that short- and medium-length chain HT esters were more effective than HT in protecting human cells (U937 and C212) from oxidative hemolysis, with HT laurate (C12) being the most effective. This derivative also exhibited better antioxidant activity than HT in the same cellular lines [99]. In addition, Candiracci et al. [100] found that among HT esters with fatty acids from C2 to C18, those with medium-sized acyl chains were the most effective in protecting human erythrocytes of healthy non-smoking volunteers. 

The synthesis of HT esters can also be performed through lipase from *Candida Antarctica*. The resulting HT esters (C4:0–C18:0) exhibited DPPH scavenging properties, inhibition of LDL oxidation and DNA protective action from hydroxy and peroxy radical-induced scission. However, the increase of fatty acid length decreased the protective effect on hydroxy-induced DNA damage [101], also reported in Grasso et al. [86]. 

To prevent undesirable alterations in the new food products [104], HT could be covalently bound to the surface of other kinds of esters, such as the polymers used in food packaging (Table 4) [105]. Fazio et al. [102] synthetized a polyacrylate containing HT (Figure 5E) named poly(hydroxytyrosyl)acrylate (PHTA) via enzymatic regioselective transesterification of the primary hydroxy group of HT with an acrylic acid methyl ester. At concentrations above 50 mg/mL, PHTA fully scavenged DPPH radicals, demonstrating a good correlation between the available phenolic groups on the polyacrylates and the radical-scavenging activity. No cytotoxic effect in RAT1 normal fibroblast cells was exhibited with the addition of polyacrylate films. 

#### 3.2.2. Ethers

Ethers, compounds produced through the esterification of its primary alcohol group with an alkyl group instead of a fatty acid, have also been prepared from HT (Figure 5B). In one study, the antioxidant activity of a family of ethers of HT in different matrices was demonstrated to follow the polar paradox theory and to decrease as the alkyl chain length increase [106] (Table 5). A working theory for this is that longer chains (C12–C18) form folded structures that shield the catechol moiety [97,99,107]. The lipophilicity was explained by the “polar paradox” theory which had already been observed in both biological and physicochemical systems for several series of antioxidant compounds [107,108]. 

#### 3.2.3. Glycosides

Another class of derivatives is based on the glucopyranoside core, a sugar-derived fragment that can have remarkable effects on pharmacological and pharmacokinetic properties and improve activity (Table 5, Figure 5F) [109]. The glycosylation reaction of HT can be carried out by biocatalytic methods with materials of animal origin. Khymenets et al. [110] reported the preparation of the β-glucuronides by human and rat liver microsomes. Trincone et al. [111] described the synthesis of α-D-glucopyranosides, α-maltosides and α-isomaltosides of HT by a visceral mass homogenate obtained from the sea hare *Aplysia fasciata*. However, these processes presented low regioselectivity, as glycosylations occurred both on primary and phenolic hydroxy groups of the aglycones [110,111]. Potocká et al. [112] reported the synthesis of HT β-fructofuranosides from sucrose by enzymatic (using yeast invertase) transglycosylations, with a yield of 19.5%. In opposition to other studies, this reaction was regioselective on the primary hydroxy group of the phenolic acceptors.

#### 3.2.4. Phospholipids

Apart from ester/ether derivatives, phosphatidyl derivatives of HT (phosphatidyl-HT) are also reported. Note that phospholipids (PLs) have amphiphilic nature and surface-active properties, making them useful as emulsifier ingredients in food [113]. The emulsifying properties of PLs also enhance the digestion and absorption of other molecules at the intestinal level, which contributes to the emulsification of lipid drops in the aqueous media, improving the formation of mixed micelles, and serving as vehicles of lipid products to the enterocytes [114]. A series of phosphatidyl-HT derivatives were synthesized (Table 5, Figure 5C), through various transphosphatidylation reactions (using phospholipase D) [115,116]. In particular, Casado et al. [115] have developed an adequate procedure and readily scalable to produce phosphatidyl-HT (PHT). Notably, this derivative was shown by the authors to exert comparable or even superior antioxidant activity than HT in diverse edible oils, as measured by the rancimat test [117]. Furthermore, Martin and coworkers [118] reported that the bioaccessibility of PHT in a micellar bile salt solution was close to 90%, confirming that after hydrolysis, its lipid products were easily solubilised within the micellar phase for intestinal absorption. Additionally, the PHT safety was confirmed in rats under acute and repeated dose oral conditions (2000 mg/kg body weight) [119].

#### 3.2.5. Isochromans

Isochromans, i.e., 3,4-dihydro-1*H*-2-benzopyran, are natural HT derivatives present in the phenolic virgin olive oil [120], possible to be obtained in high yields from HT via the Oxa-Pictet-Spengler reaction (Table 5, Figure 5D) [121]. These compounds are claimed to exert diverse bioactive properties, including inhibition of platelet aggregation [122], antioxidant and anti-inflammatory activities [123,124,125].

Hydroxy-1-aryl-isochromans, synthesized based on the reaction of HT with selected aldehydes, were shown to suppress reactive oxygen species (ROS) release from mitochondria (most likely by inactivating the formed hydrogen peroxide) from rat brain and liver, with a similar ability to that of resveratrol and much higher than trolox, *N*-acetylcysteine or melatonin [126]. Mateos et al. [127] synthesized 1,1-dimethyl-6,7-dihydroxyisochroman and 1-(3′,4′-dihydroxyphenyl)-6,7-dihydroxyisochroman via Oxa-Pictet-Spengler reaction and showed that these isochromans had high radical-scavenging activities, probably related to the number of free hydroxy moieties and free *o*-dihydroxyphenyl groups. The antioxidant capacity of these isochromans was also confirmed in bulk oils and biological systems such as brain homogenates.

#### 3.2.6. Sulphur and Selenium

Lastly, sulfation of been another way of obtaining HT derivatives, because organosulphur compounds (Table 5, Figure 5G) have antioxidant, anti-atherosclerotic, anti-proliferative, antibacterial, anti-platelet properties, as well as an ability to lower systolic blood pressure and reduce cholesterol levels [128]. Gomes et al. [129] described the sulfation of HT-acetate with two equivalents of sulphur trioxide (SO_3_), achieving a mixture of monosulfated regioisomers on the phenolic hydroxy groups with a good yield and using simple, cheap and fast procedures. A disulfated product was also obtained with eight equivalents of the sulfating agent. Furthermore, sulfate metabolites of HT [potassium 4-(2-hydroxyethyl)phenylsulfate and potassium 2-hydroxy-4-(2-hydroxyethyl)phenyl sulfate] were showed to protect intestinal cells against the pro-oxidant effect of oxidized cholesterol, with an efficiency comparable to HT. However, these chemical syntheses were not chemoselective, generating a mixture of monosulfates and disulfates, which was hard to separate [130]. Additionally, Begines et al. [131] developed a convenient, scalable, chemoselective method using the arylsulfotransferase (AST) from *Desulfitobacterium hafniense* for the sulfation of HT, and their monoacetylated derivatives. Nevertheless, it was reported that the sulfation decreased the anti-lipoperoxidant capacity, radical scavenging, and reducing properties of HT. Thus, this synthesis does not seem to be the best strategy to improve the lipophilicity of HT, meaning that the other mentioned strategies be improved.

One study also used selenium for HT funtionalisation, which is an essential element that plays a vital role as a constituent of the antioxidant enzymes glutathione peroxidase [132], thioredoxin reductase, and iodothyronine deiodinase [133]. According to Rodríguez-Gutiérrez et al. [134], HT had the ability to interact with one or two atoms of sulphur or selenium (Table 5, Figure 5H), and exerted higher inhibition of lipid peroxidation than HT in vitamin E-deficient liver microsomes rats. 

## 4. Conclusions

The nutritional and therapeutic value of OLE and HT has powered the creation of new solutions to broaden their range of applications. The big challenge of this application is to guarantee their stability and bioavailability, taking advantage of their fullest bioactivity. Undoubtedly, nanotechnology advances represent promising alternatives to incorporate these biophenols in food products. Several methods are described, including lipid-based systems, biopolymers, microorganisms and supramolecular complexes. This diversity allows the selection of the type of carrier to meet specific details of a target product or application. The main encapsulated experiments fully characterised and analysed the delivery system’s stability, while data regarding the impacts on bioaccessibility/bioavailability and bioactivity, particularly in vivo, are still very limited. In parallel, more research needs to be carried out to investigate the impact on the sensory properties of the final food product. 

Probably the main limitations of these encapsulation approaches are the difficulty of scaling-up, batch to batch reproducibility and low doses of loading. Moreover, not all methods are environmentally friendly, such as the example of the production of liposomes that often recurs to organic solvents, and ideally, other methods should be preferred. Thus, there is still space for the development of novel carriers that can fulfil all these characteristics. 

Additionally, chemical modification is proposed to overcome limitations regarding the applicability of the compounds themselves. Still, many issues remain to be elucidated also in this field. One such example is the effect of modifications on the bioaccessibility and bioavailability of the bioactive compounds, generally not elucidated by the authors. Acetylation of OLE showed to be promising in increasing its lipophilicity and bioavailability, without impact on its antioxidant capacity. In turn, the studies focusing chemical modifications on OLE are still reduced, leaving room for the development of new approaches regarding new derivatives of this biophenol. In the case of HT, esterification seems to preserve its antioxidant ability. Glycosylation, esterification with phospholipids, isochromans and sulfation were also successfully obtained, albeit this approach has diverse limitations. Probably the most evident is the use of toxic reagents in the chemical synthesis, as compared with enzymatic synthesis, thus demanding more efforts in purification. Moreover, these derivatives need additional time demanded clinical studies to be approved by FDA and EFSA to be integrated into the food industry. Notwithstanding, the stability of these chemical changes should be taken into consideration, as well as the interactions with other compounds in the final product.

## Figures and Tables

**Table 5:** Reports regarding the derivation of HT: ethers, glycosides, phospholipids, isochromans and selenium and sulphur derivatives.

**Formulation**	**Application**	**Results**	**Ref.**
Ethers			
HT ethers	Sunflower oil	↑ antioxidant activity for HT and its ethers than the commonly used antioxidants. Antioxidant activity of HT in different matrices is in agreement with the polar paradox and it is dependent of the length of the alkyls chains.	[106]
Glycosides			
HT 4′or 3′-O-β-glucuronide	Claim for food application	Methodology developed for the biocatalysed syntheses of glucuronides with a single step product isolation and a high yield.	[110]
HT α-glycosidic derivatives	Claim for food application	It was possible to glycosylate regioselectively only the alcoholic primary position (total reaction yield: 20%).	[111]
HT β-fructofuranosides	Claim for food application	Yield of fructose-transglycosylation reaction was 19.5%. The reaction was regioselective (fructosylation only on primary hydroxy group of the phenolic acceptors).	[112]
Phospholipids			
PHT	Claim for food application	Solid to solid reaction system for transphosphatidylation of phosphatidylcholine with HT.	[115,116]
PHT	Functional food	PHT antioxidant activity in diverse edible oils ≥ HT.	[117]
PHT	Claim for food application	After intestinal digestion, a closer value of EC_50_ between digested PHT and HT was achieved (0.6 and 0.5 mM respectively).	[118]
PHT	Claim for food application	PHT safe for rats and no toxicity was detected even at higher doses in both acute and repeated dose oral toxicity studies (2000 mg PHT/kg body mass).	[119]
Isochromans			
Hydroxy-1-aryl-isochromans	Claim for food application	Suppression of ROS release from mitochondria from rat brain and liver (EC_50_ of 20 µM).	[126]
Isochromans	Food preparations	Antioxidant capacity for isochromans and HT > α-tocopherol and BHT. The results partially agreed with the polar paradox.	[127]
Selenium and Sulphur			
Mono-O-sulfate HT	Claim for food application	Ten monosulfates (good yields) synthesized in 1 or 2 steps using simple, cheap and fast procedures with good yield.	[129]
Sulfate metabolites of HT	Claim for food application	Protection of intestinal cells against the pro-oxidant effect of oxidised cholesterol	[130]
Selenium and Sulphur derivatives of HT	Claim for application	Five thioureas, a disulfide, a thiol, three selenoureas, a diselenide and a selenium showed higher inhibition of lipid peroxidation than HT in vitamin E-deficient microsomes.	[134]
Sulfated HT	Claim for application	AST allowed the preparation of respective metabolites in a single step. ↓ anti-lipoperoxidant, radical scavenging and reducing properties of HT and ↑ hydrophilicity.	[131]

AST—Arylsulfotransferase; DPPH—2,2-Diphenyl-1-picrylhydrazyl; EC_50_—Half maximal effective concentration; HT—Hydroxytyrosol; PHT—phosphatidyl-HT.

## Data Availability

The data that support the findings of this study are available within the article.
